# Diet-Induced Severe Hyperhomocysteinemia Promotes Atherosclerosis Progression and Dysregulates the Plasma Metabolome in Apolipoprotein-E-Deficient Mice

**DOI:** 10.3390/nu16030330

**Published:** 2024-01-23

**Authors:** Stephen G. Andrews, Anthony M. Koehle, Devendra Paudel, Thomas Neuberger, A. Catharine Ross, Vishal Singh, Teodoro Bottiglieri, Rita Castro

**Affiliations:** 1 Department of Nutritional Sciences, Penn State University, University Park, PA 16802, USA; andrews.stephen705@gmail.com (S.G.A.); amk6684@psu.edu (A.M.K.); dzp5453@psu.edu (D.P.); acr6@psu.edu (A.C.R.); vxs28@psu.edu (V.S.); 2 Huck Institutes of the Life Sciences, Penn State University, University Park, PA 16802, USA; tun3@psu.edu; 3 Department of Biomedical Engineering, Penn State University, University Park, PA 16802, USA; 4 Center of Metabolomics, Institute of Metabolic Disease, Baylor Scott and White Research Institute, Dallas, TX 75204, USA; teodoro.bottiglieri@bswhealth.org; 5 Faculty of Pharmacy, Universidade de Lisboa, 1649-003 Lisbon, Portugal

**Keywords:** atheroma, vascular disease, hypomethylating stress, high-field MRI

## Abstract

Atherosclerosis and resulting cardiovascular disease are the leading causes of death in the US. Hyperhomocysteinemia (HHcy), or the accumulation of the intermediate amino acid homocysteine, is an independent risk factor for atherosclerosis, but the intricate biological processes mediating this effect remain elusive. Several factors regulate homocysteine levels, including the activity of several enzymes and adequate levels of their coenzymes, including pyridoxal phosphate (vitamin B6), folate (vitamin B9), and methylcobalamin (vitamin B12). To better understand the biological influence of HHcy on the development and progression of atherosclerosis, apolipoprotein-E-deficient (*apoE*^−/−^ mice), a model for human atherosclerosis, were fed a hyperhomocysteinemic diet (low in methyl donors and B vitamins) (HHD) or a control diet (CD). After eight weeks, the plasma, aorta, and liver were collected to quantify methylation metabolites, while plasma was also used for a broad targeted metabolomic analysis. Aortic plaque burden in the brachiocephalic artery (BCA) was quantified via 14T magnetic resonance imaging (MRI). A severe accumulation of plasma and hepatic homocysteine and an increased BCA plaque burden were observed, thus confirming the atherogenic effect of the HHD. Moreover, a decreased methylation capacity in the plasma and aorta, indirectly assessed by the ratio of S-adenosylmethionine to S-adenosylhomocysteine (SAM:SAH) was detected in HHD mice together with a 172-fold increase in aortic cystathionine levels, indicating increased flux through the transsulfuration pathway. Betaine and its metabolic precursor, choline, were significantly decreased in the livers of HHD mice versus CD mice. Widespread changes in the plasma metabolome of HHD mice versus CD animals were detected, including alterations in acylcarnitines, amino acids, bile acids, ceramides, sphingomyelins, triacylglycerol levels, and several indicators of dysfunctional lipid metabolism. This study confirms the relevance of severe HHcy in the progression of vascular plaque and suggests novel metabolic pathways implicated in the pathophysiology of atherosclerosis.

## 1. Introduction

Atherosclerosis and resulting cardiovascular disease (CVD) are the leading causes of death and morbidity in the US [1]. Hyperhomocysteinemia (HHcy), a condition defined by an accumulation of plasma homocysteine (Hcy), is an independent risk factor for atherosclerosis and CVD [2,3,4,5]. Nevertheless, the molecular basis of this association remains to be defined entirely [3]. Homocysteine is not naturally present in food; it is a by-product of methionine metabolism. This process begins with the conversion of methionine to S-adenosylmethionine (SAM), a universal methyl donor compound for various methyl transferase reactions [6,7,8] that causes the formation of the methylated substrate and of S-adenosylhomocysteine (SAH). Interestingly, excess SAH can competitively inhibit the activity of SAM-dependent methyltransferases. Consequently, the ratio of SAM to SAH (SAM:SAH) is commonly used and referred to as the methylation potential of the cell [4,6,7]. SAH undergoes further hydrolysis to form Hcy through a reversible reaction that thermodynamically favors SAH production when Hcy accumulates. Thus, HHcy causes a decrease in the SAM:SAH ratio, promoting hypomethylating stress. This imbalance may contribute to the observed vascular toxicity associated with high Hcy levels [4,6,7]. Hcy is then remethylated to methionine through a vitamin B9 (folate)-dependent pathway that requires vitamin B12 (cobalamin) as co-enzyme. In addition, and mainly in the liver, Hcy is remethylated to methionine by betaine, a choline metabolite. Moreover, Hcy is catabolized to cysteine by the vitamin-B6-dependent transsulfuration pathway. Thus, B vitamins play a significant role in regulating Hcy metabolism [9,10,11]. Accordingly, dietary manipulation of the content of methyl donors and B vitamins is an established approach to producing an accumulation of Hcy, especially in the presence of an excess of methionine [12,13,14].

The apolipoprotein-E-deficient (*apoE*^−/−^) mouse is a pre-clinical model of human atherosclerosis that mimics the human metabolic signature and accumulates aortic atherosclerotic plaque [15,16,17]. Thus, to better understand the biological processes related to the influence of HHcy in the development and progression of atherosclerosis, *apoE*^−/−^ mice were fed a hyperhomocysteinemic diet (HHD) (low in methyl donors and B vitamins) or a diet with adequate levels of all micronutrients (control, CD). After confirming the presence of severe HHcy in the HHD group, the volume of the aortic atheroma was quantified using high-field magnetic resonance imaging (MRI). Lastly, the plasma, aortic, and hepatic levels of intermediates of Hcy metabolism were determined, and a targeted metabolomic analysis of the plasma was conducted.

## 2. Materials and Methods

### 2.1. Animals and Diets

Seven-week-old *apoE*^−/−^ mice, purchased from Jackson Laboratory (Bar Harbor, ME, USA), were housed individually in stainless-steel wire-bottom cages in an environmentally controlled room. Only male mice were used to control for the known effect of sex hormones on atherosclerosis in this strain [18]. The animals were fed, ad libitum, water and one of the following diets that were prepared based on AIN 93G (Research Diets, New Brunswick, NJ, USA): a control diet (CD) (11 Kcal% fat, 70 Kcal% carbohydrate, 18 Kcal% protein; *n* = 8), or a HHcy diet (HHD) that had reduced levels of methyl donors and vitamins (folate, choline, vitamin B6, and vitamin B12) and an excess of methionine (*n* = 16). The composition of the CD and the altered levels of micronutrients in the HHD, shown in Table 1, were selected to match our previous studies, in which the same nutritional approach was used to induce Hcy accumulation [19,20]. Weighed portions of the diets were replaced once a week, at which time animal weights were taken. All procedures were performed in compliance with the Institutional Animal Care and Use Committee of the Pennsylvania State University, which specifically approved this study.

### 2.2. Biochemical Analyses: Blood Glucose and ALT Levels

Every two weeks, blood glucose was measured using a glucometer (Contour, Bayer, Tarrytown, NY, USA), following the manufacturer’s instructions.

Moreover, at the same time points, liver health was evaluated via the plasma alanine aminotransferase (ALT) levels (Randox AL146, Antrim, UK), following the manufacturer’s instructions.

### 2.3. Blood Collection

After 8 weeks, the mice were euthanized by carbon dioxide inhalation, and blood was collected from the portal vein into heparinized tubes and immediately placed on ice. Plasma was isolated by centrifugation at 4 °C and immediately stored at −80 °C prior to further metabolic analyses.

### 2.4. Tissue Collection

The aortas were exposed and isolated, and approximately half of the abdominal aorta was excised, immediately immersed into liquid nitrogen, and stored at −80 °C until further analysis was undertaken. The remainder of each aorta, after double perfusion (10 mL PBS followed by 10 mL neutral buffered formalin 10% (NBF, Fisher Chemical), was fixed in NBF for 24 h, and then transferred to PBS, as previously described in detail [19,20,21,22,23].

The livers were dissected, weighed, and immediately snap-frozen in liquid nitrogen or fixed in 10% NBF for 24 h, then stored in 70% ethanol until histology slides were prepared.

### 2.5. Liver Histologic Analysis

Paraffinized sections (5 mm thickness) from the fixed liver tissue were prepared by the Penn State Animal Diagnostic Laboratory and stained with hematoxylin and eosin (H&E). Images were generated using a Leica DMi8 via the LAS X 2021 software.

### 2.6. Aortic Atheroma Quantification

Magnetic resonance imaging (MRI) using an Agilent 14T microimaging system (Agilent Technologies, Inc., Santa Clara, CA, USA) was used to quantify the volume of aortic plaque burden in the brachiocephalic artery (BCA), as previously described in detail [19,20,21,23]. Scan parameters included an echo time (TE) of 13 ms, a repetition time (TR) of 100.00 ms, eight averages, a field of view (FOV) of 12.6 × 4.2 × 4.2 mm^3^, and a matrix size of 630 × 210 × 210, resulting in an isotropic resolution of 20 µm. After the acquisition, MR data were reconstructed using Matlab (The MathWorks Inc., Natick, MA, USA). Zero-filling by a factor of 2 in each direction lead to a final isotropic pixel resolution of 10 µm. Data segmentation was performed using Avizo 9.5 (Thermo Fisher Scientific, Waltham, MA, USA). The lumen of the aorta, the different plaques, and the aorta wall were manually segmented to obtain the volume of the atheroma in the vessel.

### 2.7. Quantification of Homocysteine and Methylation Metabolites

**Figure 1:**
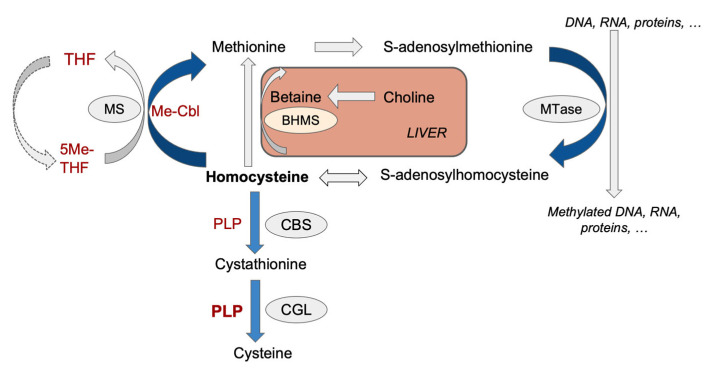
Schematic representation of the homocysteine metabolism. THF, tetrahydrofolate (vitamin B9); 5Me-THF (vitamin B9), 5-methyl THF (vitamin B9); Me-Cbl, methylcobalamine (vitamin B12), PLP, pyridoxal 5′-phosphate (vitamin B6); MTase, cellular methyltransferase; CBS, cystathionine-β-synthase; CGL, cystathionine-γ-lyase; BHMT, betaine–homocysteine methyltransferase.

Metabolites related to the methylation cycle, shown in Figure 1, were determined in the plasma, liver, and aorta tissue via liquid chromatography tandem mass spectrometry (LC-MS/MS). This included Hcy in the plasma and liver, as previously described [24,25], but not in the aorta due to a lack of sufficient tissue. Other metabolites, including methionine, SAM, SAH, betaine, choline, and cystathionine, were determined in all samples, as previously described [26,27]. All assays were performed with 2 levels of quality controls to monitor assay performance, which for all metabolites had an overall variance of <15%.

### 2.8. Targeted Metabolomic Analysis

Targeted metabolomic analysis in plasma collected at 8 weeks was performed using the commercially available MxP^®^ Quant 500 kit (Biocrates Life Sciences, Innsbruck, Austria), following the manufacturer’s instructions provided. Added to a 96-well plate were 10 μL of plasma, calibrators (7 levels), and quality controls (3 levels). The MxP^®^ Quant 500 kit can potentially identify 630 metabolites across 23 classes of compounds by LC-MS/MS. Peak identification was accomplished using Analyst 1.7.2 (SCIEX, Framingham, MA, USA) and multiple reaction monitoring. MS data were uploaded to MetIDQ (Biocrates, Innsbruck, Austria) for analyte quantitation. MetaboIndicator^TM^ (biocrates life sciences, Innsbruck, Austria) was used to calculate 232 metabolic indicators, based on the sums and/or ratios of metabolites.

### 2.9. Statistical Analysis and Bioinformatics

All analyses, except plasma metabolomics, were performed in GraphPad Prism 7 (GraphPad Software, La Jolla, CA, USA), with statistical significance set to *p* < 0.05. For the comparison of the two groups, an unpaired Student’s *t*-test was used.

For targeted metabolomic data, peak detection, concentration computation, and plate normalization were performed using MetIDQ Oxygen v3005 (Biocrates Life Sciences, Innsbruck, Austria). Metabolomic data were uploaded to and processed in MetaboAnalyst 6.0 (https://www.metaboanalyst.ca/) (last accessed on 8 January 2024) for statistical analysis (*t*-test and heatmaps). The lower limit of detection (LoDs) was calculated as 3× the median signal in the phosphate-buffered saline blanks in each plate. Analytes with more than 50% of samples below LoD in each group were filtered out, and for those with less than 50%, the <LoD values were replaced with 1/5 the minimum positive value for that variable. All data were log transformed for normalization prior to analysis.

## 3. Results and Discussion

### 3.1. General Characteristics

**Figure 2:**
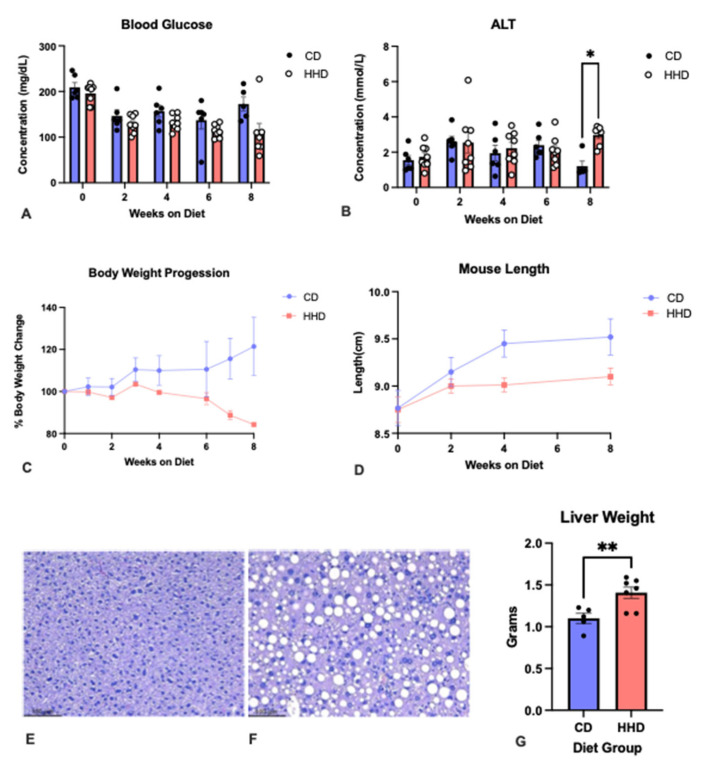
The effect of the experimental diets (HHD, hyperhomocysteinemic diet, represented in red bars; CD, control diet, represented in blue bars) on (**A**) body weight progression; (**B**) mouse length; (**C**) blood glucose; (**D**) plasma alanine transaminase, ALT; (**G**) liver weight; and (**E**,**F**) liver histology (hematoxylin-eosin-stained samples at 10× amplification) from CD and HHD mice. * *p* < 0.05; ** *p* < 0.01. All error bars = ± SEM.

Sequential measurements of body weight showed that mice fed the HHD failed to gain weight compared to the CD mice (Figure 2A) despite a similar mean calorie intake in both groups. Moreover, mice in the HHD group were smaller than the CD-fed animals (Figure 2B), evidencing slowed growth and emphasizing the essential role of micronutrients for proper development [28]. Previous studies have reported similar effects on growth when animals were fed diets with suboptimal levels of B vitamins and choline [12,14,19,20].

Next, the effect of the experimental diets on liver health was investigated. The results showed that 8 weeks of HHD feeding resulted in significantly elevated ALT levels and heavier livers than CD mice (Figure 2B and Figure 2G, respectively). Accordingly, the histology analysis revealed the HHD livers’ lipid accumulation, hepatocyte ballooning, and neutrophil infiltration, features absent in the CD mice, suggesting the development of hepatic steatosis in the first group of mice (Figure 2E,F). Dietary choline deprivation has been shown to cause fatty liver due to impaired hepatic secretion of lipids into plasma lipoproteins because of the inhibition of phosphatidylcholine (PC) synthesis from choline [29]. In addition, previous studies have also indicated that acute liver toxicity in fatty liver disease is associated with atherosclerosis and CVD [30]. Moreover, a study in which mice were fed a high-methionine–low-folate diet or a control diet reported increased Hcy concentrations and hepatic steatosis in the first group of animals [31]. Lastly, in the present study, HHD-fed mice presented a persistently lower glycemia than CD mice, which reached statistical significance after 4 weeks of the dietary intervention, a phenotype that has also been reported previously [32].

### 3.2. Homocysteine Levels and Atherosclerotic Plaque Burden

**Figure 3:**
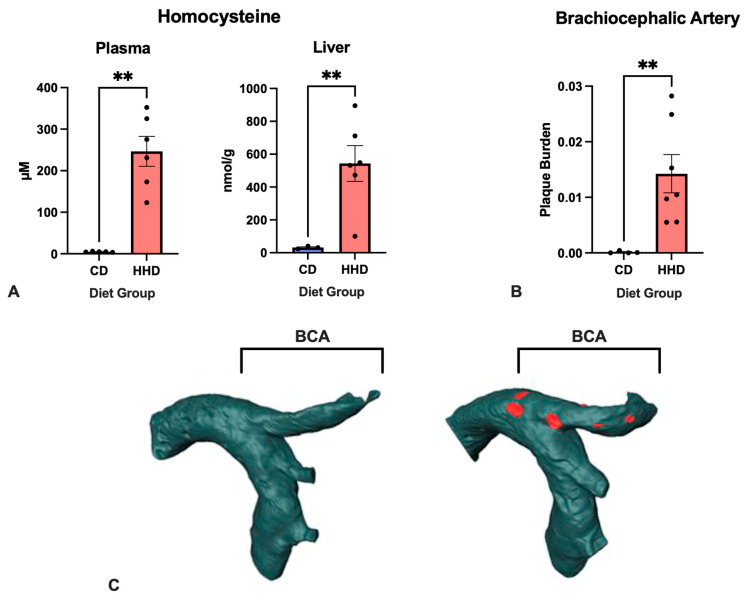
Effect of experimental diets CD (control diet) and HHD (hyperhomocysteinemic diet) on (**A**) the concentration of homocysteine in the plasma and liver; (**B**) brachiocephalic artery (BCA) atherosclerotic plaque burden. (**C**) Representative 14T-MRI images of the BCA from CD and HHD groups. Plaque burden was determined as the ratio of plaque volume (rendered in red) to wall volume. ** *p* < 0.01. All error bars = ±SEM.

The manipulation of the dietary content of choline and B vitamins is an established approach to promoting HHcy [12,13,14]. In the present study, a severe accumulation of plasma Hcy was achieved in the mice fed the HHD diet, with an average concentration of 247 ± 88.1 versus 4.7 ± 0.9 in the CD group (µM, mean ± SD) (Figure 3A). Interestingly, in our previous studies on *apoE*^−/−^ *mice*, in which diets with a similar micronutrient profile but a higher-fat content were used (40–80% fat kcal versus 5% fat kcal in the present study), we observed a milder accumulation of Hcy, suggesting that a high fat content attenuates the effect of sub-optimal levels of micronutrients on the Hcy metabolism. In liver tissue, Hcy accumulation was even more pronounced than in plasma, with mean values of 543 and 31 nmol/g for HHD and CD mice, respectively, showing that the liver was the organ in which Hcy metabolism was most affected by the HDD; this was primarily due to the combined reduced activity of cobalamin/folate-dependent, choline-dependent re-methylation into methionine and the PLP-dependent transsulfuration to cysteine.

Next, the effect of the experimental diets on atherosclerosis progression was evaluated by determining the volume of BCA aortic atheroma using 14T-MR imaging. The BCA is the segment of the aorta where the first plaques develop in *apoE^−/−^* mice [23]. As shown in Figure 3B,C, HHD mice presented significantly (*p* < 0.05) more aortic atherosclerosis burden than the CD mice. Moreover, the increased plaque burden detected in HHD BCA was accompanied by significant plaque morphology changes, such as the presence of lipid cores, luminal wall thickening, and vessel obstruction. However, the 14T-MRI imaging approach used in the present study did not address in detail features of plaque stability and necrosis, which are significant predictors of disease outcome [23].

Many studies have used dietary approaches like ours in murine models of human atherosclerosis to explore the vascular phenotype associated with HHcy. Overall, studies in which a severe (>100 µM) accumulation of plasma Hcy was achieved reported an enhanced development of atherosclerosis, supporting the results of the present study [14]. For example, Liu et al. [33] reported that eight weeks of severe HHcy aggravated aortic plaque formation. However, the findings associated with mild HHcy are inconsistent. These include our previous studies [19,20], in which, in a twelve-week feeding trial using *apoE^−/−^* mice, we could not detect significant differences at the *p* < 0.05 level for BCA plaque burden between mild hyperhomocysteinemic and control mice.

### 3.3. Methylation Indexes and the Concentrations of Relevant Metabolites in Different Tissues

**Figure 4:**
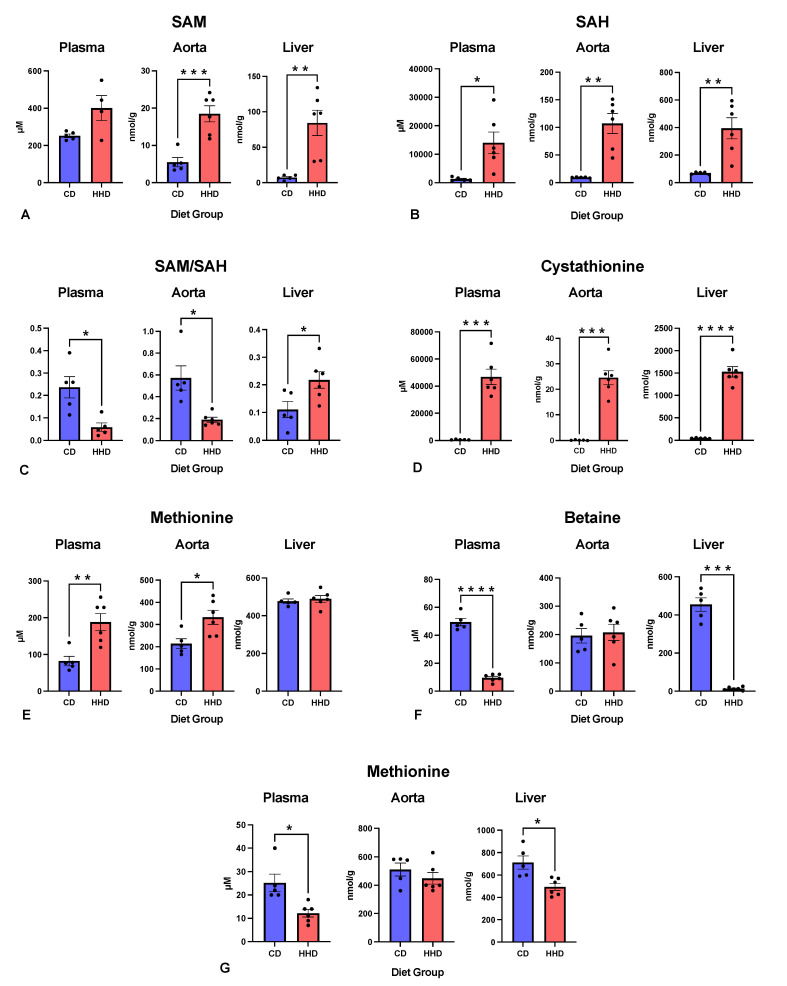
Effect of the experimental diets CD (control diet) and HHD (hyperhomocysteinemic diet) on the concentration of (**A**) SAM (S-adenosylmethionine); (**B**) SAH (S-adenosylhomocysteine); (**C**) SAM:SAH; (**D**) cystathionine; (**E**) methionine; (**F**) betaine; and (**G**) choline in the plasma, liver, and aorta; * *p* < 0.05; ** *p* < 0.01; *** *p* < 0.001; **** *p* < 0.0001. All error bars = ±SEM.

After confirming severe HHcy in the HHD group, related metabolite levels in the plasma, liver, and aortic tissue were determined (Figure 4). As anticipated, the precursor to Hcy, SAH, showed a significant elevation in all investigated tissues of HHD mice (Figure 4A). Further, the SAM:SAH ratio was significantly decreased in the plasma and aorta, indicating the presence of systemic and vascular hypomethylation in the HHD mice (Figure 4C). We have previously reported in two studies [19,20] using the same mouse model that mild HHcy was associated with systemic but not with aortic hypomethylation. This suggests that, in those studies, the levels of accumulated Hcy were insufficient to affect vascular transmethylation reactions. However, in the present study, under a severe HHcy, a significant hypomethylating environment was evident at both systemic and vascular levels. Therefore, in addition to the atherogenic effect of the HHD, we documented increased SAH and a decreased SAM:SAH ratio at the vascular level, supporting the concept that hypomethylating stress contributes to the adverse vascular consequences of HHcy [3,4,7,13,34,35,36,37,38,39]. A recent cohort study has associated elevated plasma SAH concentrations with CVD risk [40]. Plasma SAH levels were also linked to traditional CVD risk factors and subclinical atherosclerosis in individuals with low CVD risk [41]. Furthermore, plasma SAH was inversely associated with endothelial dysfunction in patients with coronary artery disease [42].

In contrast, the SAM:SAH ratio in the liver was elevated in HHD mice versus CD mice (Figure 4C). However, the excess methionine in the HHD led to a more pronounced increase in hepatic SAM than SAH (Figure 4B), compared to the plasma and aorta levels [43]. The liver is the only tissue expressing multiple isoforms of methionine-adenosyltransferase, the enzyme that converts methionine to SAM [44]. Consequently, the substantial SAM levels detected in the liver increased the SAM:SAH ratio.

The HHD notably impacted the transsulfuration pathway (Figure 4D), evident in the marked 172-fold increase in aortic cystathionine concentration, the 77-fold increase in plasma concentration, and the 31-fold increase in the liver of HHD-fed mice compared to CD mice. This substantial change across all examined tissues suggests cystathionine as a potential biomarker of hypomethylating stress. The two PLP-dependent enzymes (PLP is the active form of vitamin B6) CBS and CGL catalyze the production of cystathionine and cysteine, respectively (Figure 1). However, CGL activity is more severely affected than CBS by insufficient PLP levels [45,46]. This may be attributed to the higher affinity and increased number of PLP binding sites, with four in total, in the CBS tetramer compared to the two binding sites in the four-subunit heterodimer CGL [47]. Consequently, the HHD, which was vitamin B6 deficient, promoted a massive accumulation of cystathionine.

Finally, we determined the concentrations of betaine and its metabolic precursor, choline, in our model. The results indicated that the HHD caused a significant decrease in betaine in the liver (13 nmol/g versus 455 nmol/g in CD) (Figure 4F). Similarly, choline showed a considerable reduction (713 nmol/g in HHD versus 494 nmol/g in CD) (Figure 4G). The betaine-dependent remethylation pathway in the liver participates in homocysteine metabolism (Figure 1). As shown in Table 1, the HHD lacked choline bitartrate, which was present in the CD. Thus, the observed depletion of choline and betaine reflects its dietary origin. Additionally, the reduced betaine levels in HHD mice may have contributed to liver damage, steatosis, and the severe accumulation of Hcy, as suggested by previous studies indicating the protective role of betaine against systemic inflammation and hepatotoxicity by reducing cellular stress [48].

### 3.4. Targeted Metabolomic Analysis

**Figure 5:**
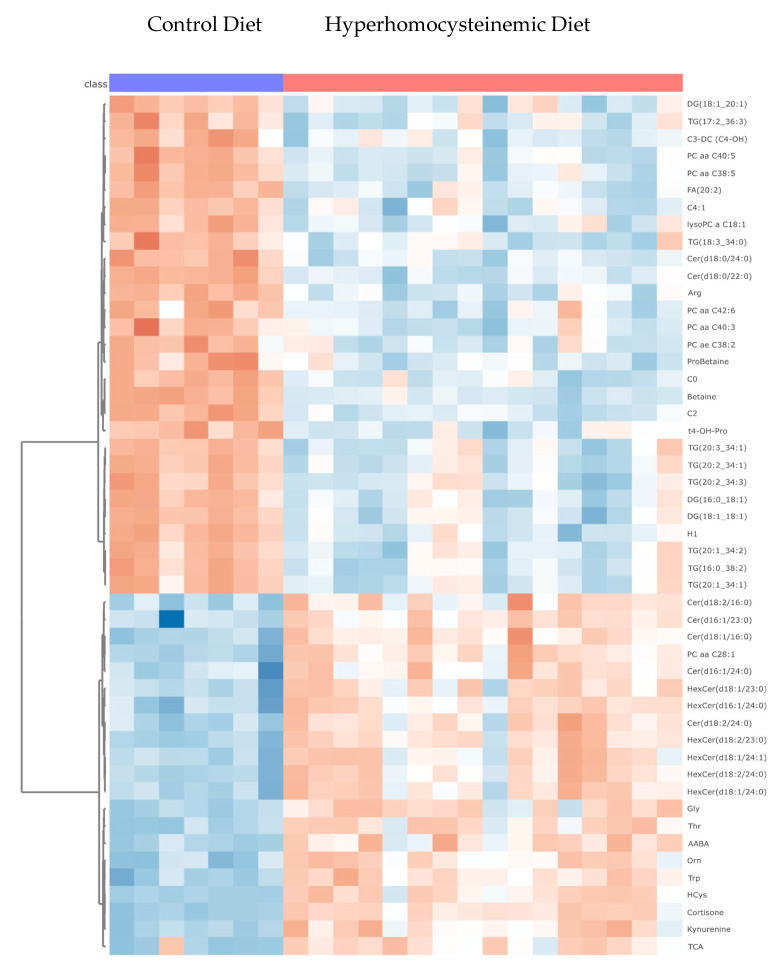
Heat map of the top 50 metabolites, including methylation specific species, ceramides (Cer), triglycerides (TG), phosphatidylcholines (PC), and amino acids, significantly altered by the experimental diets. The increasing intensity of the red color indicates higher concentrations of metabolites (max +2.3), and the increasing intensity of the blue color indicates decreased concentrations of metabolites (max −3.2).

Metabolomic profiling offers a novel approach to identifying biomarkers and studying disease mechanisms. One of the available animal models of human atherosclerosis that better mimics the human metabolite signature is the *apoE*^−/−^ mice [15]. Thus, to better understand the biological processes underlying the atherosclerotic effect of severe HHcy, we assessed the metabolomic signature in plasma from the different experimental groups. The results showed significant changes in several class compounds, seen in Table 2 and Figure 5, including acylcarnitine species, amino acids, bile acids, ceramides, sphingomyelins, fatty acids, and triacylglycerols. Several amino acids were increased in the HHD group versus the CD group, suggesting protein metabolism was affected. As explained above, the severe HHcy in the HHD-fed mice was achieved by the inadequacy of the levels of micronutrients acting as co-factors in homocysteine metabolism, including vitamin B6, a micronutrient deeply involved in amino acid metabolism and degradation. PLP, the coenzyme form of vitamin B6, participates in different catalytic functions, including transaminations, α-decarboxylations, racemizations, and the transsulfuration of Hcy to cysteine [49]. Cysteine is one of the gluthathionine constituents, a major intracellular antioxidant system. PLP has recently been suggested as a scavenger of reactive oxygen species [50,51].

**Figure 6:**
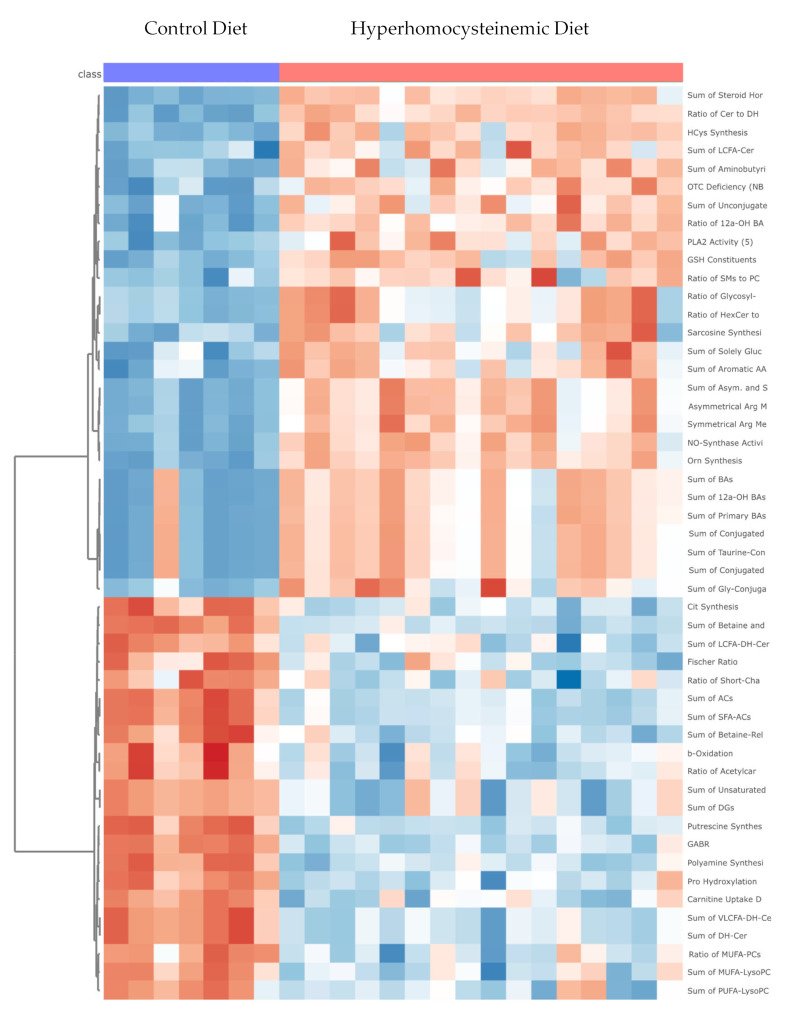
Heat map of the top 50 metabolic indicators of oxidative stress, lipid processing, fatty acid transport, bile acid production, and amino acid metabolism, significantly altered by the experimental diets. The increasing intensity of the red color indicates higher concentrations of metabolic indicators (max +2.1), and the increasing intensity of the blue color indicates decreased concentrations of metabolic indicators (max −2.2).

Additionally, the metabolomic indicators showed a various effects from HHD versus CD, as seen in Table 3 and Figure 6. These indicators are sums or sums and ratios of metabolites from the Quant 500 analysis that can reveal information on specific pathway activity and metabolic flux (Supplementary Table S1).

Acylcarnitines play an essential role in regulating lipid metabolism by permitting the transport of long-chain fatty acids into the mitochondria for β-oxidation as a source of energy [52]. A generalized depletion of plasma acylcarnitines in the HHD-fed mice was observed, suggesting a reduced β-oxidation capacity in these animals. B12 vitamin deficiency causes the accumulation of methylmalonic acid, which acts as a potent inhibitor of the rate-limiting enzyme carnitine palmitoyl transferase 1, critical for breaking long-chain fatty acids in the beta-oxidation pathway [53]. Interestingly, it has been reported that decreased plasma acylcarnitine levels in mice were associated with a defective hepatic fatty acid β-oxidation and hepatic fat deposition, thus suggesting that this may have contributed to the liver toxicity observed in the HHD group [54].

Additionally, hydrophobic bile acids, including cholic acid (CA), deoxycholic acid (DCA), and taurocholic acid (TCA), were significantly elevated in HHD mice (FDR < 0.01). Bile acids are biomarkers of lipid and carbohydrate metabolism in the liver of both mice and humans, as they are direct end products of cholesterol catabolism [46]. Alterations in bile acid metabolism are associated with vascular inflammation and atherosclerosis [55]. Moreover, bile acids, especially DCA and TCA, have been associated with liver damage [56,57,58]. Thus, the observed liver injury after 8 weeks of HHD in the present study could have contributed to the accumulation of DCA and TCA.

Moreover, in the plasma of HHD mice, the levels of 19 unsaturated ceramides and hexosyl-ceramides were significantly elevated. In contrast, four saturated ceramides decreased significantly. Ceramides are precursors to sphingolipids, and both compounds are implicated in vascular dysfunction in rodents and humans [59]. Unsaturated ceramides regulate LDL cholesterol infiltration in blood vessels, generating plaque in rodent’s vascular walls [60]. In previous studies on mice, the inhibition of ceramide synthesis limited the progression of atherosclerosis [59]. Unsaturated ceramides are known to play a substantial role in the development of cardiovascular disease. Our findings reveal a link between severe HHcy and ceramide metabolism that can influence plaque deposition, which demands further investigation.

In this study, four sphingomyelin level metabolic indicators were also significantly elevated. These include the total sum of sphingomyelins, the ratio of sphingomyelins to phosphatidylcholines, the sum of OH-deficient sphingomyelins, and the sum of long-chain fatty acid sphingomyelins. These data suggest mitochondrial dysfunction as indicated by the increase in light-chain fatty acid sphingomyelins, which have been reported to increase cardiovascular disease in humans [61]. Previous studies have suggested, however, that *apoE*^−/−^ mice have altered plasma sphingomyelin processing [62]. Other studies have reported that the pharmacologic inhibition of sphingomyelin synthesis attenuated atherosclerosis progression in *apoE*^−/−^ mice [63].

In our metabolomic analysis, many triacylglycerol species were significantly decreased in the plasma of HHD mice compared to CD. The reason needs to be clarified, and, to our knowledge, the effect of HHcy on triacylglycerol metabolism has not been previously reported. Triacylglycerols play an essential role in energy metabolism, as stores can be mobilized to produce free fatty acids converted to acetyl-CoA via β-oxidation. Acetyl-CoA then enters the citric acid cycle to generate ATP. The levels of free fatty acids (FA 18:1; 20:1 and 20:2) were also decreased in the HHD group compared to the CD one, consistent with the effect on triacylglycerols. It has been shown that tissue-specific gene hypomethylation correlates with lower plasma levels of triacylglycerols, suggesting that HHD-induced hypomethylating stress contributed to the reduction demonstrated in [64].

Lastly, the present metabolomic data suggest that phospholipase-A2 activity was increased under severe HHcy. Phospholipase-A2 converts phosphatidylcholines to lysophosphatidylcholines, which release fatty acids and eicosanoids [65]. Eicosanoids can contribute to tissue inflammation through downstream prostaglandin generation [66]. Accordingly, previous studies have shown that elevated PL-A2 is a marker of vascular inflammation [67]. As supporting evidence, the pharmaceutical inhibition of PL-A2 in *apoE*^−/−^ mice decreased vascular inflammation. In humans, increased serum levels of PL-A2 are associated with increased severity of CVD [68]. The present findings warrant further investigation into PL-A2 as a potential bridge between vascular distress and the metabolic dysfunction associated with severe HHcy [69,70].

## 4. Conclusions

In conclusion, severe HHcy promoted systemic and vascular hypomethylation and aggravated plaque formation in the BCA of *apoE*^−/−^ mice. In addition, livers from these mice exhibited hepatocellular steatosis and oxidative damage, which was supported by metabolomic changes. Moreover, substantial alterations were observed in several metabolites, including cystathionine, and various metabolomic indicators affecting lipidomic profiles. Our data demonstrate the importance of maintaining adequate levels of B vitamins to prevent HHcy-induced atherosclerosis and promote cardiovascular and liver health.

## Figures and Tables

**Table 1:** Composition of the experimental diets (CD, control diet; HHD, hyperhomocysteinemic diet).

	CD		HHD	
	gm	Kcal	gm	Kcal
Casein	180	720	180	720
Corn Starch	431	1725	431	1725
Maltodextrin 10	155	620	155	620
Sucrose	100	400	100	400
Cellulose	35	0	35	0
Primex ^1^	25	225	25	225
Corn Oil	25	225	25	225
Mineral Mix S10001	35	0	35	0
Vitamin Mix V10001 ^2^	10	40	0	0
Vitamin Mix V14904 ^3^	0	0	10	40
L-Cystine	3	12	3	12
L-Methionine	0	0	3.1	12
Choline bitartrate	2.5	0	0	0
Pyridoxine HCl (×10^3^)	0	0	0.2	0
Folic acid (×10^3^)	0	0	0.1	0
Cyanocobalamin, 0.1% (×10^−6^)	0	0	2	0
Succinylsulfathiazole	8.6	0	8.6	0

^1^ The typical Primex composition is shown in the Supplementary File S1; ^2^ With adequate levels of all vitamins; ^3^ Without vitamin B6, B9, or B12.

**Table 2:** Number of significantly altered plasma metabolite concentrations between CD (control diet) and HHD (hyperhomocystenemic) mice.

Metabolite Class		Potential	Detected	FDR (<0.05) (CD vs. HHD)	
		Metabolites	Metabolites	Increased	Decreased
LC-MS/MS Metabolites	Alkaloids	1	0	0	0
	Amine Oxides	1	0	0	0
	Amino Acids	20	20	13	4
	Amino Acids Related	30	24	7	8
	Bile Acids	14	12	12	0
	Biogenic Amines	9	7	0	0
	Carboxylic Acids	7	4	0	0
	Cresols	1	1	0	0
	Fatty Acids	12	9	0	3
	Hormones	4	1	1	0
	Indoles and Derivatives	4	3	1	0
	Nucleobases Related	2	0	0	0
	Sugars	1	1	0	1
	Vitamins and Cofactors	1	1	0	1
FIA-MS/MS metabolites	Acylcarnitines	40	13	0	8
	Glycerophospholipids	90	87	3	26
	*(Lysophosphatidylcholines & Phosphatidylcholines)*				
	Sphingolipids	15	10	10	0
	Cholesterol Esters	22	21	0	4
	Ceramides	28	28	19	4
	Dihydroceramides	8	4	0	0
	Glyceroceramides	34	34	17	0
	*(Mono-*, *Di-*, *and Trihexosylceramindes)*				
	Diacylglycerols	44	20	0	16
	Triacylglycerols	242	237	0	214
	Total	630	537	83	289

LC-MS/MS: liquid chromatography tandem mass spectrometry; FIA-MS/MS: Flow injection analysis tandem mass spectrometry.

**Table 3:** Number of significantly altered metabolomic indicators (indexes based on relative metabolite concentrations) between CD (control diet) and HHD (hyperhomocysteinemic diet)-fed mice.

	FDR (<0.05) CD vs. HHD			Relevant to Inflammation and Atherosclerosis
Metabolic Indicator Class	Significant	Increased	Decreased	
Amino Acids	13	9	4	1
Amino Acid Related	18	11	7	4
Acylcarnitines	13	2	11	1
Bile Acids	15	13	2	0
Biogenic Amines	4	1	3	1
Carboxylic Acids	1	1	0	0
Ceramides	14	9	5	1
Cholesteryl Esters	1	0	1	1
Diglycerides	4	1	4	1
Fatty Acids	5	2	3	1
Hormones	1	1	0	0
Indole and Derivatives	2	1	1	0
Lysophosphatidylcholines	5	1	4	0
Phosphatidylcholines	5	2	3	3
Sphingomyelins	4	4	0	0
Triacylglycerols	4	0	4	2

## Data Availability

Data are contained within the article and Supplementary Materials.

## Supplementary Materials

The following supporting information can be downloaded at: https://www.mdpi.com/article/10.3390/nu16030330/s1, Supplementary Table S1: List of metabolic indicators included in the MxP Quant 500 assay. The indicators were calculated by MetIDQ Oxygen v3005 software using Metabo *INDICATOR* formulas. Supplementary File S1: Typical Primex composition provided by Research Diets.
