# Supplementary material for: Diet-Induced Severe Hyperhomocysteinemia Promotes Atherosclerosis Progression and Dysregulates the Plasma Metabolome in Apolipoprotein-E-Deficient Mice
Source: Nutrients. 2024 Jan 23;16(3):330. doi: 10.3390/nu16030330 (PMC10856797; doi:10.3390/nu16030330)
Supplement: Supplementary file 1 [file nutrients-16-00330-s001.zip › Supplementary file S1 .pdf]

STRATAS FOODS

Technical Data Sheet

Product Code - 101650

50# Primex Z All-Purpose Shortening

Benefits and Applications:

- Designed specifically for applications where bland flavor and light color
- Excellent creaming properties
- Excellent donut frying capabilities
- 0 grams of trans fat per tablespoon (12g)

Kosher:
Parve

Handling and Storage:
Care must be taken during storage and shipment to avoid damaging the flavor. Shortening, no matter how carefully packaged, will pick up flavors if stored near items giving off strong odors. Suggested storage 65-75°F.

Shelf Life:
Shelf life 1 year from date of manufacture

Packaging:
50# cube: 12 per tier; 4 tiers; 48 per pallet (corrugated outer case with a poly liner)
Pallets are stretch wrapped for extra cleanliness and extra stability during shipment.

Cubic Measurements: (width x length x height)
50# cube: 11.25" x 13.125" x 13.25"; 1912.97 cu. inches; 1.11 cu. ft.

Ingredients:
Palm Oil, Hydrogenated Soybean Oil

Allergens:
None

Analytical:

Color, Lovibond Red	1.5 Max
Free Fatty Acids, %	0.07 Max
Peroxide Value, meq/kg	1.0 Max
Mettler Dropping Point °C	42.2 - 44.4
Trans Fatty Acids, %	2.25 Max

STRATAS FOODS

Nutritional Data Sheet
100gram

Product Name: 50# Primex Z All-Purpose Shortening
Product Code: 101650

This nutritional information is provided based on typical composition of the product and the data for individual products may vary from this information. This material is provided for informational purposes only and not as a specification. The total fat percentage is expressed as triglyceride equivalents and the fatty acid percentages are expressed as free fatty acids. The total on the individual fatty acids may not equal total fat percentage due to the exclusion of the glycerol backbone in the free fatty acid calculation. This complies with the definition of fat and fatty acids in title 21 Code of Federal Regulations section 101.9.

Information below provided on a 100 gram basis

Calories:	884
Protein:	0 g
Total Carbohydrates:	0 g
Sodium:	0 mg
Vitamin A:	0 IU
Cholesterol:	0 mg
Fat:	100 g
Saturated:	50.3 g
Trans Fatty Acids:	1.9 g
cis-Monounsaturated:	36.1 g
cis-Polyunsaturated :	7.3 g

Nutrition Facts
Serving Size 1 Tablespoon (12g)
Servings Per Container varies

Amount Per Serving	
**Calories** 110	Calories from Fat 110
	% Daily Value*
**Total Fat** 12g	**18%**
Saturated Fat 6g	**30%**
Trans Fat 0g	
Polyunsaturated Fat 1g	
Monounsaturated Fat 4.5g	
**Cholesterol** 0mg	**0%**
**Sodium** 0mg	**0%**
**Total Carbohydrate** 0g	**0%**
**Protein** 0g	

Not a significant source of dietary fiber, sugars, vitamin A, vitamin C, calcium, and iron.

* Percent Daily Values are based on a 2,000 calorie diet.

Version Date: 02/21/2014; Version: 2

This product does not require a Material Safety Data sheet to be in compliance with OSHA regulations. It is a food-grade product which is intended for edible uses. It is not a health, safety or toxic hazard. In addition, it is subject to the Federal Food, Drug and Cosmetic Act. PLEASE NOTE: This specification is provided for information purposes only and should not be relied upon as a basis for product performance. It is suggested you evaluate the product on at least a laboratory basis prior to its commercial usage. This specification may be superseded by a later issue. Please consult you sales representative to confirm you have the correct specification.

NO WARRANTIES OF MERCHANTABILITY OR FITNESS FOR A SPECIFIC USE OR PURPOSE, EXPRESS OR IMPLIED, ARE MADE. These specifications are not intended to and shall not be construed to be instructions or suggestions for use which may be in violation of valid patent rights. Stratas Foods LLC 7130 Goodlett Farms Parkway, Suite 200, Memphis, TN 38016 (888)404-1004

STRATAS FOODS

Typical Fatty Acid Composition for Primex, 101650

Fatty Acid	%	Typical Value
C6:0	%	0.0
C8:0	%	0.0
C10:0	%	0.0
C12:0	%	0.4
C14:0	%	1.1
C14:1	%	0.0
C15:0	%	0.0
C15:1	%	0.0
C16:0	%	42.2
C16:1	%	0.0
C17:0	%	0.0
C17:1	%	0.0
C18:0	%	7.10
C18:1 Trans	%	0.40
C18:1	%	37.8
C18:2 Trans	%	0.2
C18:2	%	10.0
C18:3 Trans	%	0.1
C18:3	%	0.3
C20:0	%	0.4
C20:1	%	
C22:0	%	
C22:1	%	
C24:0	%	
Other	%	
Total	%	100.0
SAFA	%	51.2
cis-PUFA	%	10.3
cis-MONO	%	37.8
Total TFA	%	0.7
